# Nutritional Processing Quality of Sika Deer (*Cervus nippon*) Venison in Different Muscles

**DOI:** 10.3390/foods13223661

**Published:** 2024-11-17

**Authors:** Chunai Jin, Songhuan Cui, Yushun Lu, Zhiman Li, Xiaohui Huo, Yanbo Wang, Jiyue Sha, Yinshi Sun

**Affiliations:** 1 Institute of Special Wild Economic Animals and Plants, Chinese Academy of Agriculture Sciences, Changchun 130112, China; kimchunai@163.com (C.J.); shcui71@126.com (S.C.); luyushun@caas.cn (Y.L.); lzm091215@163.com (Z.L.); xiaohui.114@163.com (X.H.); wangyanbo20231735@163.com (Y.W.); 2 College of Chinese Medicinal Materials, Jilin Agricultural University, Changchun 130118, China

**Keywords:** sika deer (*Cervus nippon*) venison, nutritional processing quality, different muscles, amino acid, fatty acid

## Abstract

In order to investigate the nutritional processing quality of sika deer (*Cervus nippon*) venison at different sites, the pH_24 h_, tenderness, pressurized water loss rate, meat color, intramuscular fat, moisture, protein, amino acid, fatty acid and squalene contents of sika deer venison were determined in twelve sites: foreleg, hind leg, outer tenderloin, rump, neck meat, chest meat, deer flank, abdominal rib, high rib, tenderloin, anterior tendon and posterior tendon. The results showed that the pH_24 h_ of sika deer venison at different sites was 5.49~5.78; the tenderness of outer tenderloin (31.71 N) was the lowest, and the neck meat (68.53 N) was the highest; the squeezing moisture of tenderloin (28.12%) was the largest, and the foreleg (12.34%) was the smallest; the brightness of outer tenderloin L* (29.68) was the lowest, and the redness a* and yellowness b* of deer flank were the highest; the intramuscular fat and moisture were 0.66~4.97% and 71.00~73.78%, respectively; and the protein content of outer tenderloin (23.44%) and rump (24.02%) was high. The venison meat contained 17 kinds of amino acids, and the total amount was 63.87~79.33 g/100 g. It was rich in essential amino acids, mainly lysine and leucine, accounting for 64.29~65.39% of non-essential amino acids, which was close to the ideal protein composition. Palmitoleic acid and oleic acid were the main monounsaturated fatty acids in venison, and the contents of abdominal ribs were the highest, 16,875.33 mg/kg and 31,772.73 mg/kg, respectively. The contents of essential fatty acids were also the highest in abdominal ribs (11,225.37 mg/kg); forelegs, hind legs, outer tenderloins, rumps, neck meat, chest meat, high rib, tenderloins, anterior tendons and posterior tendons were all good sources of polyunsaturated fatty acids. Squalene content was highest in the abdominal rib (100.85 mg/kg). The nutritional processing quality of sika deer venison in different muscles is significantly different, and this study can provide a data basis for the evaluation and processing of sika deer venison quality.

## 1. Introduction

As a high-value meat product, venison is rich in protein, amino acids and polyunsaturated fatty acids and has a low cholesterol content, which is one of the important animal food resources [1]. Among all cervids, venison is in greatest demand, with a market value estimated at over USD 1.5 billion. As the most developed deer industry country in the world, about 75% of the income of the deer industry in New Zealand comes from deer meat, which has broad market prospects in the United States, Europe and China [2]. The sika deer (*Cervus nippon*) is a rare special livestock breed in China, which has been recorded in both Famous Doctor Bie Lu and Compendium of Materia Medica. The sika deer venison has good nutrition and health care functions and has a unique taste and aroma [3], popular with consumers.

Meat quality and acceptability often depend on its physicochemical properties, particularly color and fat content. Consumers usually judge the freshness of products by meat color, followed by flavor characteristics, and tenderness and juiciness are also key to affecting the purchase rate [4]. Amino acids are important determinants of venison flavor and taste, and free amino acids can participate in the formation of flavor substances through the Maillard reaction [5]. The content and composition of fatty acids depend on genetic and nutritional factors and can influence oxidative stability, flavor and even nutritional quality [6]. Fatty acids include saturated and unsaturated fatty acids, and excessive intake of saturated fatty acids leads to increased triglyceride and low-density lipoprotein levels and increases the risk of obesity, diabetes, and coronary heart disease. In contrast, unsaturated fatty acids reduce cholesterol and triglyceride content, thereby reducing the risk of cardiovascular disease and improving immune function [7]. In addition, squalene, a 30-hydrocarbon nonsteroidal intermediate in cholesterol biosynthesis, prevents lipid peroxidation on human skin surfaces and confers some radioprotection in mice receiving lethal whole-body radiation doses [8]. Studies have shown that the nutritional composition and physicochemical characteristics of venison meat vary greatly depending on the site, further affecting its flavor characteristics [9,10]. Due to the market demand for higher quality livestock meat products increasing significantly, as well as the product quality characteristics and health and nutritional quality concerns, analyzing the nutrient composition of different sika deer meat muscles is crucial for grading its product quality.

The aim of this study was to compare the nutritional processing quality of different muscles of sika deer and to compare and analyze the meat quality traits, amino acids, free fatty acids and squalene contents of twelve muscles of sika deer, providing an important reference for the quality evaluation and processing of sika deer.

## 2. Materials and Methods

### 2.1. Materials and Reagents

**Figure 1:**
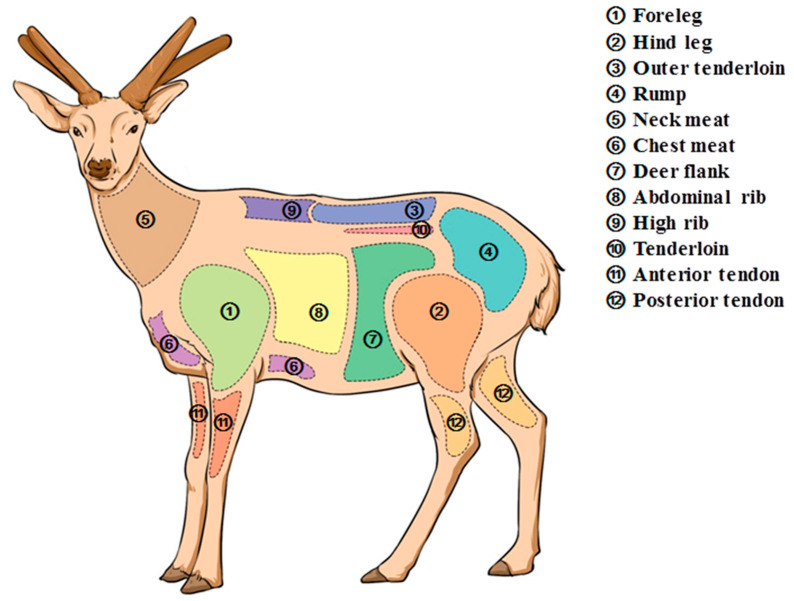
Cuts from the carcass.

The sika deer (*Cervus nippon*) venison samples were obtained from five 4-year-old male sika deer with similar body weight and qualified quarantine from the Zuojia Antler Breeding Base, Institute of Special Products, Chinese Academy of Agricultural Sciences (Jilin City, China). Deer meat was divided into 12 muscles: foreleg, hind leg, outer tenderloin, rump, neck meat, chest meat, deer flank, abdominal rib, high rib, tenderloin, anterior tendon and posterior tendon, according to Figure 1. Surface tendons, tendons, membranes and fats were removed, partly for mass determination and partly prepared into dry samples for determination of nutritional indicators. Mixed standards of 18 amino acids (S000029) and chromatographically pure ninhydrin were purchased from Sykam (Munich, Germany). Mixed standards of 37 fatty acids (LRAD3869) were purchased from Merck (Darmstadt, Germany). Chromatographically pure methanol and n-hexane were purchased from Fishser (Pittsburgh, PA, USA). Ultrapure water with resistivity ≥ 18.25 MΩ was prepared by laboratory ultrapure water mechanism.

### 2.2. Meat Quality Determination

#### 2.2.1. pH_24 h_

The pH_24 h_ was measured using the pH-STAR pH meter (Beijing Tianxiang Feiyu Technology Co., Ltd., Beijing, China), and the mean value was taken in triplicate.

#### 2.2.2. Tenderness

According to previous studies [11,12], the meat samples with a central temperature of 0~4 °C were heated at 80 °C in a thermostatic water bath. When the central temperature of the meat samples reached 70 °C, the meat samples were taken out and cooled to the central temperature as the initial temperature. Cutting meat samples were drilled 2.5 cm along the direction parallel to the muscle fibers with a circular sampler with a diameter of 1.27 cm using a C-LM3B tenderness instrument (Northeast Agricultural University, Harbin, China), and shear force values were recorded and averaged over three replicates.

#### 2.2.3. Pressurized Water Loss Rate

The circular meat column 1 cm thick and 2.5 cm in diameter was weighed along the vertical direction of muscle fibers, wrapped by double-layer gauze and 18-layer qualitative filter paper, and pressurized on a RH-1000 meat strain hydraulic tester (Guangzhou Runhu Instruments Co., Ltd., Guangzhou, China) for 35 kg for 5 min. The gauze was removed, and the paper was filtered and weighed again; the pressurized water loss rate was measured, and the mean value was taken in triplicate.

#### 2.2.4. Flesh Color

The meat block was cut with a thickness of no less than 2 cm along the vertical direction of muscle fiber and placed flat in the tray. The lens of the CR 400 color difference instrument (Konica Minolta (China) Investment Co., Ltd., Shanghai, China) was vertically clenched to the meat surface. The brightness (L*), redness (a*) and yellowness (b*) values were measured and recorded. The mean values were taken in triplicate.

### 2.3. Nutrient Determination

The fat and water content in venison meat were determined by Soxhlet extraction and distillation, respectively, and protein content was determined by Kjeltec 8400 automatic Kjeldahl apparatus (Shanghai Teiyue International Trading Co., Ltd., Shanghai, China) using Kjeltec nitrogen determination method.

### 2.4. Amino Acids

#### 2.4.1. Amino Acids Assay

An amount of 100 mg of samples was weighed, 10 mL HCL (6 mol/L) was added to freeze for 3~5 min, dried at 110 °C ± 1 °C constant temperature, and taken out and cooled after hydrolysis for 22~24 h. Then, 0.5 mL of filtrate was taken and evaporated under vacuum, adding 1 mL of sample diluent and shaken well, filtered with 0.22 μm filter membrane, and determined with S-433D amino acid automatic analyzer (Sykam Co., Ltd., Free State of Bavaria, Munich, Germany).

The operating conditions are as follows: buffer A is sodium citrate buffer solution with an equivalent concentration of 0.12 N (pH 3.45), buffer B is sodium citrate buffer solution with an equivalent concentration of 0.20 N (pH 10.85), derivatization solution is ninhydrin solution, eluent flow rate is 0.45 mL/min, reactor temperature is 130 °C, column temperature is 58 °C, injection volume is 50 μL, the UV detection wavelength of proline was 440 nm, and the rest were 570 nm.

#### 2.4.2. Amino Acid Score

The measured essential amino acids were converted into milligrams of amino acids per gram of protein, and the nutritional value was assessed according to the optimal matching model of the Food and Agriculture Organization of the United Nations (FAO)/World Health Organization (WHO), and the amino acid score (AAS) was calculated by the formula:

AAS/% = (Amino Acid Content Tested)/(Content of isoamino acids in FAO/WHO model) × 100%

### 2.5. Fatty Acid Assay

An amount of 0.03 g of uniform test sample was weighed, 100 mg pyrogallic acid and 2 mL 95% ethanol solution were added and mixed well, and 10 mL hydrochloric acid solution was added and hydrolyzed in a water bath at 70–80 °C for 40 min. It was then cooled to room temperature, 10 mL of 95% ethanol solution and 50 mL of petroleum ether mixture were added and shaken for 5 min, and the ether layer extract was collected after standing. After volatilization overnight, 4 mL of n-hexane was added to dissolve, shaken for 30 s and allowed to stand; sodium hydrogen sulfate was added to precipitate the salt, filtered with a 0.22 μm filter membrane, and measured using a 7000D/8890 gas chromatography–mass spectrometer (Agilent Co., Ltd., Santa Clara, CA, USA).

The GC-MS conditions gas phase conditions were as follows: inlet 280 °C, column model DB-FastFame 30 m × 250 µM × 0.25 µM, column flow rate 1 mL/min, transfer line temperature 280 °C, oven heating program—initial 80 °C, hold 0.5 min; gradient I rate 40 °C/min, to 165 °C, hold 1 min; gradient II rate 4 °C/min, to 230 °C, hold 4 min. The post-run temperature was 260 °C, the time was 5 min, and the acquisition time was 23 min. The mass spectrometric conditions were as follows: electron energy 70 eV; solvent delay time 2 min; run time 1 min; and the rest were set according to the qualitatively obtained retention time, fragment ion mass-to-charge ratio, and peak appearance order.

### 2.6. Squalene Assay

An amount of 0.1 g of the sample was weighed, 10 mL of hydrochloric acid (8.3 mol/L) was added, and acid hydrolysis was performed in a water bath at 80 °C ± 1 °C. After cooling, 10 mL of 95% ethanol was added, fat was extracted with petroleum ether mixture, dried with nitrogen, and 8 mL of 2% potassium hydroxide/methanol solution was added and saponified in an 80 °C ± 1 °C water bath. After cooling, 7 mL of 14% boron trifluoride methanol solution was added, methyl esterified in 80 °C ± 1 °C water bath, and quickly cooled to room temperature. An amount of 15 mL of n-hexane was added and mixed well; then, saturated sodium chloride solution was added and allowed to separate. Five milliliters of upper liquid was obtained and dehydrated with anhydrous sodium sulfate before testing.

The GC conditions were as follows: The chromatographic column was Rtx^®^-XLB (30 m × 0.35 mm × 0.5 μm, Reiscon Technologies, Cambridge, MA, USA), the injection port temperature was 250 °C, the FID detector temperature was 280 °C, the hydrogen flow rate was 30 mL/min, and the air flow rate was 300 mL/min. The programmed temperature conditions were as follows: the initial temperature was 80 °C and held for 0.3 min, then increased to 300 °C at 60 °C/min and held for 15 min. The carrier gas was high-purity nitrogen (purity ≥ 99.999%); the constant flow mode was a flow rate of 1.0 mL/min; the injection mode was a split injection, with a split ratio of 5:1; and the injection volume was 1 μL.

### 2.7. Statistical Analysis

To assess reproducibility, each assay was performed in triplicate. Experimental data were presented as mean ± S.D., the one-way ANOVA was performed using SPSS 26.0 software, and Duncan’s method was used for multiple comparisons to determine their significance. Different lowercase letters indicate significant differences at different sites (*p* < 0.05).

## 3. Results and Discussion

### 3.1. Nutritional Processing Quality of Sika Deer Venison in Different Muscles

In order to compare the nutritional processing quality of sika deer venison at different sites, the meat pH_24 h_, meat tenderness, squeezing moisture, meat color, intramuscular fat, moisture and protein content were determined. As an important index for the quality evaluation of poultry meat, pH can directly affect meat quality, color and flavor [11]. As shown in Table 1, the pH_24 h_ values of sika deer venison at 12 sites ranged from 5.49 to 5.78. Among them, the pH_24 h_ of rumps (5.49) was lower, and the pH_24 h_ of anterior tendon and abdominal rib was 5.78 and 5.72, respectively, which was significantly higher than that of other sites (*p* < 0.05). Similar to previous findings [9,13,14], sika deer meat was weakly acidic, with pH_24 h_ values generally similar to pork and slightly lower than beef, which may be related to metabolic differences resulting from fiber type composition in meat. Studies have shown that glycogen decomposes into glucose after slaughter in beef, pork, and other poultry, and lactic acid is produced after glycolysis, resulting in a decrease in pH [15]. These results suggest that there may be more glycolytic fibers in rumps than in other sites, while the glycogen content required for glycolysis is higher in anterior tendons and abdominal ribs.

Meat tenderness is the key to the palatability and quality of meat [16]. The tenderness of sika deer venison at different sites was the lowest outer tenderloin (31.71 N), the meat was tender, and the tenderness of neck meat (68.53 N) was significantly higher than that at other sites (*p* < 0.05), and it required more chewing. Water holding capacity is one of the reasons affecting tenderness and juiciness of meat products, and most of the water in meat is stored in myofibrils and muscle fibers and their spaces [17]. Sika deer venison had the largest press moisture tenderloin value (28.12%) and the smallest foreleg value (12.34%), indicating that sika deer foreleg had tighter muscle structure and stronger water-holding ability.

Sika deer venison had the lowest brightness L* outer tenderloin (29.68) and the highest chest meat (34.95); deer flank and abdominal rib redness a* were 21.98 and 20.95, respectively, slightly higher than other muscles; yellowness b* was higher in deer flank and tenderloin, 8.26 and 8.17, respectively. Meat color is the key characteristic of the salability of fresh meat. In this study, the brightness of the outer tenderloin was low, and the meat color of the deer flank and abdominal rib was dark. Differences in venison brightness at different sites may be due to light scattering changes caused by differences in surface muscle fiber structure, and meat color differences are also associated with differences in the concentrations of pigmentation, myoglobin and hemoglobin [18,19].

Consumer sensory evaluation scores were positively correlated with intramuscular fat content of meat [20,21,22]. Therefore, increasing intramuscular fat content to improve meat quality is a major concern in breeding efforts in the meat industry [23]. The content of intramuscular fat in venison was 0.66~4.97%, and the content of the flank and abdominal rib was 4.29% and 4.97%, respectively. The higher moisture content was found in the foreleg (73.78%) and slightly lower in rumps (71.00%) and deer flank (71.36%). Protein content was higher in outer tenderloins and rumps, 23.44% and 24.02%, respectively, which were higher than in sirloin of Wagyu, Wangus and Angus-by-Charolais or Limousine [24]. The protein content of sika deer venison meat at different sites ranged from 18.92% to 24.02%, which was similar to the commercially available beef, Karakul sheep and commercially available pork [25,26,27] and belonged to high-protein livestock meat.

### 3.2. Amino Acid Content in Different Muscles of Sika Deer Venison

Amino acids, as basic constituents of proteins, influence the nutritional value and flavor characteristics of meat [28,29]. According to Table 2, there were 17 amino acids in the venison of sika deer at different sites, except cystine. The total amino acid content of sika deer venison meat at different sites ranged from 63.87~79.33 g/100 g, of which the anterior tendon, foreleg and posterior tendon were higher, 79.33, 79.24 and 78.89 g/100 g, respectively, and the abdominal rib (63.87 g/100 g) and deer flank (68.11 g/100 g) were significantly lower than other sites (*p* < 0.05). According to nitrogen balance and growth needs, amino acids can be divided into essential and non-essential amino acids [30]. Compared with the amino acid composition of western roe deer, European red deer, Dalin antelope and domestic beef [31,32,33], the essential amino acid composition of sika deer venison at different sites was higher in lysine and leucine, 6.03~7.67 g/100 g and 6.18~7.61 g/100 g, respectively. Among them, lysine content was higher in the anterior tendon, foreleg and posterior tendon (7.67, 7.65 and 7.63 g/100 g, respectively), and leucine content was higher in the foreleg (7.61 g/100 g). It has been shown that both lysine and leucine, as important components of muscle growth, stimulate protein synthesis in skeletal muscle by activating the rapamycin signaling pathway [34,35]. This was followed by valine, threonine and isoleucine, with methionine having the lowest content (0.33~0.66 g/100 g). Among the non-essential amino acid components, glutamic acid had the highest content (10.74~13.68 g/100 g), followed by aspartic acid (6.22~7.91 g/100 g), and both amino acids had positive effects on intestinal health, muscle protein synthesis, and body immunity [36,37]. Glutamate content was higher in the anterior tendon (13.68 g/100 g), and aspartate content was higher in the foreleg (7.91 g/100 g). According to FAO/WHO demonstration criteria, essential amino acids/total amino acids should account for about 40% of the better protein composition [38]. The content of essential amino acids in sika deer venison accounted for 39.13~39.54% of the total amino acids and 64.29~65.39% of the non-essential amino acids, indicating that there was no significant difference in the ratio of essential to non-essential amino acids in sika deer venison at different sites, which was close to the ideal protein composition.

### 3.3. Content of Taste Amino Acids in Different Muscles of Sika Deer Venison

The type and content of amino acids directly affect the specific taste, flavor and aroma formation of meat [39]. Taste amino acids can be divided into umami amino acids (aspartate and glutamic acid), sweet amino acids (threonine, serine, glycine, and alanine), bitter amino acids (histidine, leucine, isoleucine, valine, tyrosine, arginine, methionine, and phenylalanine), and other odorless amino acids [40]. Table 3 shows the comparison of tasty amino acid contents in sika deer venison. The results showed that the tasty amino acid content in sika deer venison was higher in specific muscles. The contents of umami amino acids in venison ranged from 16.96 to 21.50 g/100 g, and the contents of the foreleg and anterior tendon were 21.50 and 21.54 g/100 g, respectively. Studies have shown that amino acid mixtures or combinations of amino acids with other substances increase the umami taste of meat more than individual amino acids [5]. The contents of sweet and bitter amino acids in sika deer venison were higher in the foreleg (11.17 g/100 g), anterior tendon (11.25 g/100 g) and posterior tendon (11.18 g/100 g), respectively. The contents of taste amino acids in the flank and abdominal ribs of deer were significantly lower than those in other muscles (*p* < 0.05), and the taste amino acids in the venison of sika deer at different muscles were significantly different.

### 3.4. Essential Amino Acid Proportion and Amino Acid Score of Sika Deer Venison at Different Sites

Dietary protein quality is mainly characterized by its essential amino acid content. Essential amino acids cannot be produced endogenously and must be obtained exogenously from food or other forms. They are considered essential amino acids in the human diet. According to the pattern of essential amino acids in ideal protein and amino acids in whole egg protein proposed by FAO/WHO, the amino acid scores of sika deer meat at different sites were calculated to evaluate its nutritional value. The results showed that the mass fractions of isoleucine, leucine, lysine, phenylalanine + tyrosine, threonine and valine in the total amino acids of sika deer venison exceeded the reference values of the pattern spectrum (Table 4). Most of the energy in the human diet comes from cereals, but the limitation of lysine content in cereals makes them low-quality proteins [41]. However, lysine was higher (171.60~176.32%) in sika deer venison amino acid score, and neck meat (176.32%) was significantly higher than outer tenderloin (172.42%), rump (172.85%), deer flank (172.99%), abdominal rib (171.60%) and tenderloin (172.45%) (*p* < 0.05). It was found that sika deer venison meat is rich in natural lysine and has the potential to supplement the deficiency of cereals and promote normal development and calcium absorption [42]. In addition, the amino acid scores of sika deer venison at each site were significantly different in isoleucine, phenylalanine + tyrosine, threonine and valine. The isoleucine score within the tenderloin (123.23%) was higher, significantly higher than neck meat (120.47%) and abdominal ribs (120.35%) (*p* < 0.05). Phenylalanine + tyrosine and threonine also had high tenderloin scores (123.23%) of 133.22% and 127.26%, respectively. However, the valine scores of the outer tenderloin, rump and tenderloin were similar, 106.66%, 106.45% and 106.54%, respectively, which were significantly higher than those of the foreleg (103.46%), neck meat (103.58%) and anterior tendon (102.95%) (*p* < 0.05). The results showed that sika deer venison could balance the contents of many amino acids in the mixed diet.

### 3.5. Fatty Acid Content in Different Muscles of Sika Deer Venison

The biological value of fat depends mainly on the amount and type of fatty acids contained in it. The results showed that the total fatty acid contents in the flank and abdominal ribs of sika deer were 102,903.55 mg/kg and 116,949.90 mg/kg, respectively, which were significantly higher than those in other muscles (*p* < 0.05) (Table 5). The content of saturated fatty acids was also higher in the flank and abdominal ribs of deer, 45,433.36 mg/kg and 51,594.45 mg/kg, respectively, and the proportion of palmitic acid and stearic acid was greater. This is consistent with previous studies that palmitic and stearic acids are the most prominent saturated fatty acids in red meat [43], but excessive intake may influence the development of vascular and coronary artery disease. Palmitoleic acid and oleic acid were the main components of monounsaturated fatty acids in sika deer venison, of which palmitoleic acid content was highest in the abdominal rib (16,875.33 mg/kg) and lowest in the tenderloin (2542.52 mg/kg); oleic acid content was highest in the abdominal rib (31,772.73 mg/kg) and lowest in rumps (4109.46 mg/kg). Polyunsaturated fatty acids have the potential to reduce cholesterol levels and risk of cardiovascular disease [44]. The content of polyunsaturated fatty acids in the venison of sika deer at different sites was the highest in abdominal ribs (14,631.23 mg/kg) and the lowest in neck meat (5295.21 mg/kg). Similar to the results of polyunsaturated fatty acids in elk meat and venison [45,46], linoleic acid was the highest proportion of polyunsaturated fatty acids in venison meat of sika deer at all sites, with the abdominal rib content (11,028.78 mg/kg) significantly higher than that in the outer tenderloin (4258.24 mg/kg), rump (3829.20 mg/kg), neck meat (3572.59 mg/kg) and posterior tendon (5426.19 mg/kg) (*p* < 0.05).

### 3.6. Essential Fatty Acid Index of Sika Deer Venison at Different Sites

Most of the polyunsaturated fatty acids are essential fatty acids (EFAs) that cannot be synthesized by the human body, mainly including ω-3 series polyunsaturated fatty acids (α-linolenic acid, eicosapentaenoic acid, docosahexaenoic acid) with α-linolenic acid as the mother, and ω-6 series unsaturated fatty acids (linoleic acid, γ-linolenic acid, eicosatetraenoic acid) with linoleic acid as the mother. Studies have shown that dietary supplementation with EFAs reduces metabolic and health disorders. Eicosapentaenoic acid and docosahexaenoic acid can exert hypolipidemic effects by inhibiting the resynthesis of triglycerides in the intestinal wall and liver [41]. Docosahexaenoic acid, as an important structural component of neural cell membranes, also has a positive effect on depression and stress [47]. The content of EFA (linoleic acid + α-linolenic acid) in venison of sika deer at different sites was the highest in the abdominal rib (11,225.37 mg/kg) (Table 6), which was significantly higher than that in the outer tenderloin (4268.59 mg/kg), rump (3841.74 mg/kg), neck meat (3594.95 mg/kg) and posterior tendon (5483.62 mg/kg) (*p* < 0.05). However, EFA accounted for 11.09% of its total fatty acids in the abdominal ribs, which was significantly lower than that in the foreleg (21.10%), posterior leg (18.28%), rump (17.38%), tenderloin (23.27%), anterior tendon (25.52%) and posterior tendon (23.01%) (*p* < 0.05). Among them, the content of ω-3 series polyunsaturated fatty acids was the highest in the abdominal rib (264.21 mg/kg) and the lowest in the outer tenderloin (19.33 mg/kg); ω-6 series unsaturated fatty acids were the highest in the abdominal rib (14,008.12 mg/kg) and the lowest in the neck meat (5031.62 mg/kg). The ratio of polyunsaturated fatty acid content between omega-6 and omega-3 series ranged from 40.92 to 346.11, with the highest ω-6/ω-3 value in the outer tenderloin, followed by rumps (278.51) and neck meat (120.79), and the lowest in the hind legs, with large differences. According to WHO recommendations [48], the recommended proportion of PUFA/SFA in the human diet is 0.4 or higher. The PUFA to SFA ratio of sika deer venison at different sites ranged from 0.26 to 1.16, of which the foreleg (0.69), hind leg (0.66), outer tenderloin (0.53), rump (0.73), neck meat (0.45), chest meat (0.50), high rib (0.56), tenderloin (0.84), anterior tendon (1.16) and posterior tendon (1.10) met the criteria and were good sources of polyunsaturated fatty acids.

### 3.7. Squalene Content in Venison of Different Muscles of Sika Deer

Squalene, as a known natural antioxidant, helps to reduce cholesterol and triglyceride concentrations in serum and inhibits the development of various cancers such as colon, lung, and skin cancers, and is abundant in deep-sea shark liver and olive oil [49]. In this study, squalene was found to be present in sika deer venison (Table 7), and the content of squalene in venison at different sites was highest in the abdominal rib (100.85 mg/kg), followed by deer flank (93.25 mg/kg) and chest meat (74.14 mg/kg), which were significantly higher than those in the outer tenderloin (34.63 mg/kg) and neck meat (30.13 mg/kg) (*p* < 0.05). Liu reported that the contents of squalene in biceps femoris, longissimus dorsi and buffalo biceps femoris were 5.982 mg/kg, 2.099 mg/kg and 25.77 mg/kg, respectively [50], and the squalene contents in each part of venison were much higher than those in the above beef in this study.

## 4. Conclusions

In this study, the nutritional processing quality of sika deer venison meat at different sites was analyzed by measuring the meat quality, nutritional composition, amino acids, fatty acids and squalene contents in 12 sites: foreleg, hind leg, outer tenderloin, rump, neck meat, chest meat, deer flank, abdominal rib, high rib, tenderloin, anterior tendon and posterior tendon. The results showed that the pH_24 h_ value of venison was 5.49~5.78, the neck meat required the most chewing strength; the water-holding ability of the foreleg was stronger, and the water content was higher; the redness and intramuscular fat content of deer flank and abdominal rib were higher; the protein content of outer tenderloin and rump was higher; and the venison meat of sika deer belonged to high-protein livestock meat. The essential amino acids of venison are complete, lysine and leucine (essential amino acids), as well as glutamic acid and aspartic acid (non-essential amino acids), are the main amino acids, and the amino acids in the anterior tendon, foreleg and posterior tendon are the most abundant and close to the ideal protein composition. Deer flank and abdominal ribs have higher fatty acid content. Monounsaturated fatty acids were mainly composed of palmitoleic acid and oleic acid, both of which were highest in the abdominal ribs. The content of EFA (linoleic acid + *α*-linolenic acid) in venison of sika deer at different sites was also the highest in abdominal ribs. The *ω*-6/*ω*-3 values were highest in the outer tenderloins, followed by rumps and neck meat. The foreleg, hind leg, outer tenderloin, rump, neck meat, chest meat, high rib, tenderloin, anterior tendon and posterior tendon all met the recommended criteria for PUFA/SFA for the human diet as recommended by the WHO and are good dietary sources of polyunsaturated fatty acids. The content of squalene in venison was highest in abdominal ribs, followed by deer flank and chest meat. This study can provide data for the quality assessment and processing of sika deer venison and provide favorable information for consumers focusing on nutrition and health to select venison products to further develop the venison market. The underlying molecular mechanisms of venison meat in the future still need to be explored to identify factors underlying meat quality differences associated with muscles.

## Figures and Tables

**Table 1:** Nutritional processing qualities of sika deer venison in different muscles.

Location	pH_24 h_	Tenderness (N)	Press Moisture (%)	Color L*	Color a*	Color b*	Intramuscular Fat (%)	Water (%)	Protein (%)
Foreleg	5.65 ± 0.07 ^abcd^	51.70 ± 7.17 ^b^	12.34 ± 4.19 ^d^	30.75 ± 2.34 ^bcd^	17.28 ± 1.08 ^c^	5.63 ± 0.51 ^b^	0.86 ± 0.20 ^c^	73.78 ± 0.59 ^a^	21.67 ± 0.59 ^ab^
Hind leg	5.51 ± 0.16 ^cd^	44.73 ± 6.44 ^bc^	22.82 ± 6.17 ^abc^	30.16 ± 3.16 ^cd^	15.81 ± 2.74 ^c^	6.00 ± 1.42 ^b^	0.98 ± 0.30 ^c^	72.80 ± 0.52 ^ab^	22.73 ± 0.92 ^ab^
Outer tenderloin	5.54 ± 0.03 ^bcd^	31.71 ± 7.01 ^c^	23.98 ± 6.17 ^ab^	29.68 ± 2.24 ^d^	16.30 ± 0.96 ^c^	5.82 ± 0.64 ^b^	1.20 ± 0.21 ^bc^	71.66 ± 1.16 ^ab^	23.44 ± 1.03 ^a^
Rump	5.49 ± 0.14 ^d^	40.25 ± 11.44 ^bc^	24.77 ± 8.98 ^ab^	32.25 ± 4.50 ^abcd^	16.88 ± 1.88 ^c^	6.87 ± 1.89 ^ab^	1.18 ± 0.35 ^bc^	71.00 ± 3.93 ^b^	24.02 ± 3.70 ^a^
Neck	5.66 ± 0.17 ^abcd^	68.53 ± 13.86 ^a^	15.23 ± 5.02 ^bcd^	31.28 ± 1.57 ^abcd^	17.99 ± 1.96 ^bc^	5.67 ± 1.40 ^b^	1.60 ± 0.38 ^bc^	73.26 ± 0.45 ^ab^	20.94 ± 0.94 ^ab^
Chest	5.60 ± 0.05 ^abcd^	53.56 ± 9.72 ^b^	20.07 ± 5.55 ^abcd^	34.95 ± 4.05 ^a^	17.93 ± 2.75 ^bc^	6.68 ± 2.07 ^ab^	2.25 ± 0.86 ^b^	72.42 ± 0.83 ^ab^	21.92 ± 0.49 ^ab^
Flank	5.63 ± 0.07 ^abcd^	52.26 ± 10.92 ^b^	21.09 ± 12.24 ^abcd^	34.21 ± 1.79 ^abc^	21.98 ± 2.22 ^a^	8.26 ± 1.54 ^a^	4.29 ± 1.71 ^a^	71.36 ± 2.42 ^b^	20.85 ± 1.98 ^ab^
Abdominal rib	5.72 ± 0.11 ^a^	44.09 ± 16.73 ^bc^	24.50 ± 9.07 ^ab^	34.21 ± 1.11 ^abc^	20.95 ± 2.07 ^a^	7.37 ± 1.31 ^ab^	4.97 ± 1.86 ^a^	72.14 ± 1.67 ^ab^	18.92 ± 1.62 ^b^
High rib	5.69 ± 0.05 ^ab^	48.33 ± 13.18 ^b^	17.67 ± 3.83 ^bcd^	32.04 ± 1.69 ^abcd^	20.70 ± 2.09 ^ab^	7.38 ± 0.89 ^ab^	1.84 ± 0.64 ^bc^	72.92 ± 0.85 ^ab^	20.82 ± 2.59 ^ab^
Tenderloin	5.52 ± 0.11 ^cd^	38.09 ± 9.01 ^bc^	28.12 ± 4.74 ^a^	34.75 ± 2.38 ^ab^	20.21 ± 1.41 ^ab^	8.17 ± 0.85 ^a^	0.95 ± 0.23 ^c^	72.74 ± 1.50 ^ab^	20.69 ± 5.26 ^ab^
Anterior tendon	5.78 ± 0.18 ^a^	51.27 ± 15.21 ^b^	13.22 ± 5.72 ^cd^	30.09 ± 3.59 ^cd^	17.91 ± 2.88 ^bc^	6.91 ± 2.40 ^ab^	0.66 ± 0.25 ^c^	73.26 ± 0.49 ^ab^	20.37 ± 4.89 ^ab^
Posterior tendon	5.67 ± 0.15 ^abc^	50.08 ± 9.49 ^b^	17.61 ± 5.68 ^bcd^	30.35 ± 3.20 ^cd^	17.98 ± 1.16 ^bc^	6.05 ± 0.57 ^b^	0.81 ± 0.16 ^c^	72.93 ± 0.38 ^ab^	22.48 ± 1.06 ^ab^

Values represent the mean ± S.D., *n* = 5. Data in the same column are marked with different letters indicating significant differences (*p* < 0.05).

**Table 2:** Amino acids content of sika deer venison in different muscles.

Amino Acid	Content (g/100 g)											
	Foreleg	Hind Leg	Outer Tenderloin	Rump	Neck	Chest	Flank	Abdominal Rib	High Rib	Tenderloin	Anterior Tendon	Posterior Tendon
Aspartate (Asp)	7.91 ± 0.32 ^a^	7.73 ± 0.13 ^abc^	7.85 ± 0.18 ^ab^	7.74 ± 0.09 ^abc^	7.43 ± 0.24 ^bc^	7.42 ± 0.25 ^bc^	6.71 ± 0.67 ^d^	6.22 ± 0.55 ^e^	7.35 ± 0.18 ^c^	7.71 ± 0.14 ^abc^	7.85 ± 0.20 ^ab^	7.79 ± 0.16 ^abc^
Threonine (Thr) *	3.97 ± 0.16 ^a^	3.89 ± 0.06 ^ab^	3.93 ± 0.08 ^ab^	3.89 ± 0.04 ^ab^	3.77 ± 0.13 ^ab^	3.72 ± 0.11 ^b^	3.39 ± 0.31 ^c^	3.16 ± 0.30 ^d^	3.71 ± 0.10 ^b^	3.87 ± 0.07 ^ab^	3.96 ± 0.07 ^a^	3.96 ± 0.06 ^a^
Serine (Ser)	3.33 ± 0.11 ^a^	3.23 ± 0.05 ^ab^	3.26 ± 0.07 ^ab^	3.23 ± 0.06 ^ab^	3.15 ± 0.12 ^ab^	3.11 ± 0.11 ^b^	2.86 ± 0.27 ^c^	2.67 ± 0.23 ^d^	3.10 ± 0.08 ^b^	3.21 ± 0.07 ^ab^	3.31 ± 0.08 ^a^	3.29 ± 0.07 ^ab^
Glutamate (Glu)	13.59 ± 0.56 ^ab^	12.78 ± 0.22 ^bcd^	12.83 ± 0.24 ^bcd^	12.73 ± 0.19 ^cd^	12.93 ± 0.43 ^abcd^	12.34 ± 0.49 ^d^	11.53 ± 1.10 ^e^	10.74 ± 0.98 ^f^	12.66 ± 0.35 ^cd^	12.78 ± 0.23 ^bcd^	13.68 ± 0.73 ^a^	13.42 ± 0.34 ^abc^
Glycine (Gly)	3.66 ± 0.11 ^abc^	3.54 ± 0.07 ^bcd^	3.54 ± 0.07 ^bcd^	3.53 ± 0.04 ^bcd^	3.64 ± 0.30 ^abc^	3.51 ± 0.18 ^cd^	3.31 ± 0.15 ^ef^	3.20 ± 0.17 ^f^	3.40 ± 0.14 ^de^	3.41 ± 0.06 ^de^	3.74 ± 0.13 ^ab^	3.79 ± 0.13 ^a^
Alanine (Ala)	4.98 ± 0.16 ^ab^	4.85 ± 0.09 ^abc^	4.90 ± 0.09 ^abc^	4.85 ± 0.05 ^abc^	4.89 ± 0.22 ^abc^	4.69 ± 0.15 ^c^	4.37 ± 0.25 ^d^	4.25 ± 0.35 ^d^	4.69 ± 0.13 ^c^	4.78 ± 0.08 ^bc^	5.02 ± 0.12 ^ab^	5.04 ± 0.09 ^a^
Cystine (Cys)	0.52 ± 0.18	0.49 ± 0.17	0.49 ± 0.18	0.50 ± 0.18	0.54 ± 0.19	0.48 ± 0.16	0.41 ± 0.16	0.43 ± 0.18	0.51 ± 0.19	0.47 ± 0.16	0.53 ± 0.19	0.50 ± 0.19
Valine (Val) *	4.10 ± 0.19 ^ab^	4.08 ± 0.12 ^abc^	4.16 ± 0.12 ^a^	4.10 ± 0.07 ^ab^	3.92 ± 0.09 ^bcd^	3.89 ± 0.09 ^cd^	3.58 ± 0.26 ^e^	3.39 ± 0.27 ^f^	3.88 ± 0.10 ^d^	4.05 ± 0.03 ^abcd^	4.08 ± 0.06 ^abc^	4.11 ± 0.05 ^ab^
Methionine (Met) *	0.51 ± 0.13 ^abc^	0.56 ± 0.14 ^ab^	0.53 ± 0.12 ^abc^	0.57 ± 0.09 ^ab^	0.44 ± 0.09 ^abc^	0.43 ± 0.17 ^bc^	0.45 ± 0.21 ^abc^	0.33 ± 0.21 ^c^	0.47 ± 0.11 ^abc^	0.47 ± 0.07 ^abc^	0.66 ± 0.15 ^a^	0.56 ± 0.22 ^ab^
Isoleucine (Ile) *	3.83 ± 0.22 ^ab^	3.75 ± 0.08 ^abc^	3.84 ± 0.11 ^ab^	3.78 ± 0.04 ^abc^	3.65 ± 0.09 ^bc^	3.60 ± 0.12 ^c^	3.29 ± 0.26 ^d^	3.08 ± 0.28 ^e^	3.61 ± 0.10 ^c^	3.75 ± 0.06 ^abc^	3.86 ± 0.10 ^ab^	3.87 ± 0.10 ^a^
Leucine (Leu) *	7.61 ± 0.46 ^a^	7.31 ± 0.13 ^abc^	7.45 ± 0.30 ^abc^	7.29 ± 0.11 ^abc^	7.21 ± 0.21 ^abc^	7.02 ± 0.25 ^c^	6.45 ± 0.49 ^d^	6.18 ± 0.55 ^d^	7.07 ± 0.27 ^bc^	7.30 ± 0.20 ^abc^	7.53 ± 0.22 ^ab^	7.45 ± 0.28 ^abc^
Tyrosine (Tyr)	2.77 ± 0.12 ^a^	2.66 ± 0.08 ^a^	2.71 ± 0.09 ^a^	2.69 ± 0.06 ^a^	2.60 ± 0.11 ^a^	2.58 ± 0.09 ^a^	2.34 ± 0.30 ^b^	2.14 ± 0.22 ^c^	2.56 ± 0.09 ^a^	2.66 ± 0.06 ^a^	2.72 ± 0.10 ^a^	2.72 ± 0.09 ^a^
Phenylalanine (Phe) *	3.54 ± 0.16 ^a^	3.43 ± 0.08 ^abc^	3.46 ± 0.10 ^ab^	3.40 ± 0.06 ^abc^	3.38 ± 0.10 ^abc^	3.26 ± 0.10 ^c^	3.01 ± 0.24 ^d^	2.87 ± 0.24 ^d^	3.30 ± 0.06 ^bc^	3.42 ± 0.05 ^abc^	3.51 ± 0.09 ^a^	3.49 ± 0.05 ^a^
Lysine (Lys) *	7.65 ± 0.31 ^a^	7.33 ± 0.11 ^ab^	7.39 ± 0.12 ^ab^	7.32 ± 0.10 ^ab^	7.34 ± 0.19 ^ab^	7.04 ± 0.19 ^b^	6.48 ± 0.58 ^c^	6.03 ± 0.50 ^d^	7.17 ± 0.20 ^b^	7.22 ± 0.13 ^b^	7.67 ± 0.28 ^a^	7.63 ± 0.21 ^a^
Histidine (His)	2.89 ± 0.15 ^de^	3.23 ± 0.24 ^bc^	3.58 ± 0.18 ^a^	3.33 ± 0.17 ^ab^	2.80 ± 0.27 ^de^	3.03 ± 0.20 ^cd^	2.59 ± 0.34 ^e^	2.26 ± 0.29 ^f^	2.84 ± 0.25 ^de^	3.00 ± 0.14 ^cd^	2.79 ± 0.11 ^de^	2.92 ± 0.10 ^d^
Arginine (Arg)	5.38 ± 0.26 ^a^	5.19 ± 0.10 ^ab^	5.24 ± 0.12 ^ab^	5.20 ± 0.06 ^ab^	5.01 ± 0.25 ^b^	5.03 ± 0.18 ^b^	4.63 ± 0.47 ^c^	4.31 ± 0.33 ^d^	4.98 ± 0.13 ^b^	5.19 ± 0.08 ^ab^	5.36 ± 0.24 ^a^	5.30 ± 0.20 ^ab^
Proline (Pro)	3.00 ± 0.09 ^ab^	2.86 ± 0.05 ^bc^	2.84 ± 0.06 ^cd^	2.83 ± 0.06 ^cd^	2.99 ± 0.16 ^ab^	2.80 ± 0.12 ^cd^	2.70 ± 0.14 ^de^	2.60 ± 0.15 ^e^	2.79 ± 0.10 ^cd^	2.81 ± 0.06 ^cd^	3.05 ± 0.11 ^a^	3.05 ± 0.06 ^a^
Total amino acid (TAA)	79.24 ± 3.30 ^a^	76.91 ± 0.99 ^ab^	77.99 ± 1.81 ^ab^	76.99 ± 0.71 ^ab^	75.71 ± 2.35 ^ab^	73.96 ± 2.39 ^b^	68.11 ± 5.76 ^c^	63.87 ± 5.43 ^d^	74.10 ± 2.14 ^b^	76.10 ± 1.27 ^ab^	79.33 ± 2.33 ^a^	78.89 ± 1.65 ^a^
EAA/TAA, %	39.39 ± 0.52	39.46 ± 0.42	39.43 ± 0.24	39.42 ± 0.34	39.25 ± 0.20	39.17 ± 0.51	39.13 ± 0.31	39.19 ± 0.15	39.42 ± 0.36	39.54 ± 0.22	39.41 ± 0.22	39.38 ± 0.30
EAA/NEAAs, %	65.00 ± 1.41	65.19 ± 1.15	65.10 ± 0.67	65.09 ± 0.93	64.62 ± 0.54	64.40 ± 1.38	64.29 ± 0.83	64.45 ± 0.41	65.06 ± 0.99	65.39 ± 0.60	65.04 ± 0.59	64.98 ± 0.83

* Essential amino acids. Values represent the mean ± S.D., *n* = 5. Data in the same column are marked with different letters indicating significant differences (*p* < 0.05).

**Table 3:** Flavor amino acid content of sika deer venison in different muscles.

Flavor Amino Acid	Content (g/100 g)											
	Foreleg	Hind Leg	Outer Tenderloin	Rump	Neck	Chest	Flank	Abdominal Rib	High RiB	Tenderloin	Anterior Tendon	Posterior Tendon
Umami	21.50 ± 0.87 ^a^	20.50 ± 0.31 ^abc^	20.68 ± 0.41 ^abc^	20.47 ± 0.25 ^abc^	20.36 ± 0.67 ^abc^	19.76 ± 0.72 ^c^	18.24 ± 1.76 ^d^	16.96 ± 1.53 ^e^	20.01 ± 0.51 ^bc^	20.49 ± 0.38 ^abc^	21.54 ± 0.91 ^a^	21.21 ± 0.47 ^ab^
Sweet taste	15.94 ± 0.48 ^ab^	15.50 ± 0.24 ^abc^	15.62 ± 0.29 ^abc^	15.50 ± 0.16 ^abc^	15.46 ± 0.63 ^abc^	15.03 ± 0.54 ^c^	13.93 ± 0.88 ^d^	13.28 ± 1.03 ^d^	14.91 ± 0.44 ^c^	15.27 ± 0.27 ^bc^	16.03 ± 0.28 ^ab^	16.07 ± 0.19 ^a^
Bitter taste	30.63 ± 1.56 ^ab^	30.21 ± 0.47 ^abc^	30.96 ± 0.93 ^a^	30.37 ± 0.22 ^abc^	29.02 ± 0.96 ^bc^	28.84 ± 0.88 ^bc^	26.34 ± 2.39 ^d^	24.56 ± 2.20 ^e^	28.70 ± 0.79 ^c^	29.84 ± 0.43 ^abc^	30.51 ± 0.84 ^ab^	30.43 ± 0.72 ^abc^
Odorless	11.17 ± 0.43 ^a^	10.69 ± 0.23 ^ab^	10.73 ± 0.31 ^ab^	10.65 ± 0.21 ^ab^	10.87 ± 0.40 ^ab^	10.33 ± 0.35 ^b^	9.60 ± 0.77 ^c^	9.07 ± 0.72 ^c^	10.48 ± 0.46 ^b^	10.50 ± 0.26 ^b^	11.25 ± 0.44 ^a^	11.18 ± 0.40 ^a^

Values represent the mean ± S.D., *n* = 5. Data in the same column are marked with different letters indicating significant differences (*p* < 0.05).

**Table 4:** Proportion of essential amino acids and amino acid score of sika deer venison in different muscles.

Location	Project	Ile	Leu	Lys	Met + Cys	Phe + Tyr	Thr	Val
	Amino acid pattern	4.0	7.0	5.5	3.5	6.0	4.0	5.0
Foreleg	Fraction of total amino acid mass (%)	4.84 ± 0.10 ^ab^	9.60 ± 0.24	9.66 ± 0.15 ^ab^	1.30 ± 0.30	7.96 ± 0.09 ^ab^	5.02 ± 0.04 ^bc^	5.17 ± 0.10 ^bc^
	Amino acid score, AAS (%)	120.93 ± 2.39 ^ab^	137.09 ± 3.40	175.65 ± 2.65 ^ab^	37.13 ± 8.54	132.64 ± 1.57 ^ab^	125.38 ± 1.06 ^bc^	103.46 ± 1.90 ^bc^
Hind leg	Fraction of total amino acid mass (%)	4.88 ± 0.07 ^ab^	9.50 ± 0.14	9.54 ± 0.15 ^abc^	1.37 ± 0.40	7.91 ± 0.07 ^ab^	5.05 ± 0.02 ^ab^	5.31 ± 0.12 ^ab^
	Amino acid score, AAS (%)	122.04 ± 1.81 ^ab^	135.72 ± 2.04	173.40 ± 2.72 ^abc^	39.02 ± 11.53	131.89 ± 1.12 ^ab^	126.30 ± 0.45 ^ab^	106.20 ± 2.40 ^ab^
Outer tenderloin	Fraction of total amino acid mass (%)	4.92 ± 0.08 ^ab^	9.54 ± 0.20	9.48 ± 0.15 ^c^	1.30 ± 0.33	7.92 ± 0.07 ^ab^	5.03 ± 0.03 ^abc^	5.33 ± 0.12 ^a^
	Amino acid score, AAS (%)	122.99 ± 1.98 ^ab^	136.35 ± 2.82	172.42 ± 2.68 ^c^	37.27 ± 9.44	131.93 ± 1.19 ^ab^	125.84 ± 0.81 ^abc^	106.66 ± 2.44 ^a^
Rump	Fraction of total amino acid mass (%)	4.91 ± 0.08 ^ab^	9.47 ± 0.13	9.51 ± 0.12 ^bc^	1.39 ± 0.32	7.91 ± 0.07 ^ab^	5.05 ± 0.02 ^ab^	5.32 ± 0.13 ^a^
	Amino acid score, AAS (%)	122.75 ± 2.04 ^ab^	135.32 ± 1.85	172.85 ± 2.18 ^bc^	39.80 ± 9.19	131.81 ± 1.10 ^ab^	126.29 ± 0.52 ^ab^	106.45 ± 2.61 ^a^
Neck	Fraction of total amino acid mass (%)	4.82 ± 0.08 ^b^	9.53 ± 0.15	9.70 ± 0.06 ^a^	1.30 ± 0.32	7.90 ± 0.13 ^ab^	4.98 ± 0.08 ^cd^	5.18 ± 0.09 ^bc^
	Amino acid score, AAS (%)	120.47 ± 1.96 ^b^	136.10 ± 2.15	176.32 ± 1.11 ^a^	37.04 ± 9.10	131.71 ± 2.23 ^ab^	124.58 ± 1.95 ^cd^	103.58 ± 1.82 ^bc^
Chest	Fraction of total amino acid mass (%)	4.87 ± 0.08 ^ab^	9.49 ± 0.15	9.53 ± 0.14 ^abc^	1.23 ± 0.39	7.90 ± 0.08 ^ab^	5.03 ± 0.04 ^abc^	5.26 ± 0.08 ^abc^
	Amino acid score, AAS (%)	121.73 ± 1.93 ^ab^	135.55 ± 2.10	173.23 ± 2.54 ^abc^	35.15 ± 11.03	131.66 ± 1.32 ^ab^	125.68 ± 0.88 ^abc^	105.29 ± 1.63 ^abc^
Flank	Fraction of total amino acid mass (%)	4.83 ± 0.08 ^ab^	9.47 ± 0.26	9.51 ± 0.13 ^bc^	1.25 ± 0.47	7.85 ± 0.15 ^ab^	4.98 ± 0.06 ^cd^	5.26 ± 0.10 ^abc^
	Amino acid score, AAS (%)	120.87 ± 2.01 ^ab^	135.32 ± 3.78	172.99 ± 2.38 ^bc^	35.63 ± 13.36	130.90 ± 2.45 ^ab^	124.45 ± 1.41 ^cd^	105.22 ± 1.95 ^abc^
Abdominal rib	Fraction of total amino acid mass (%)	4.81 ± 0.06 ^b^	9.68 ± 0.25	9.44 ± 0.08 ^c^	1.19 ± 0.52	7.86 ± 0.15 ^ab^	4.94 ± 0.06 ^d^	5.31 ± 0.07 ^ab^
	Amino acid score, AAS (%)	120.35 ± 1.52 ^b^	138.23 ± 3.55	171.60 ± 1.43 ^c^	34.04 ± 14.73	130.96 ± 2.53 ^ab^	123.44 ± 1.49 ^d^	106.11 ± 1.39 ^ab^
High rib	Fraction of total amino acid mass (%)	4.87 ± 0.04 ^ab^	9.54 ± 0.14	9.68 ± 0.13 ^ab^	1.32 ± 0.34	7.91 ± 0.10 ^ab^	5.01 ± 0.03 ^bc^	5.24 ± 0.07 ^abc^
	Amino acid score, AAS (%)	121.66 ± 1.08 ^ab^	136.29 ± 2.02	175.97 ± 2.30 ^ab^	37.67 ± 9.66	131.91 ± 1.72 ^ab^	125.21 ± 0.63 ^bc^	104.73 ± 1.44 ^abc^
Tenderloin	Fraction of total amino acid mass (%)	4.93 ± 0.06 ^a^	9.59 ± 0.17	9.48 ± 0.08 ^c^	1.24 ± 0.20	7.99 ± 0.06 ^a^	5.09 ± 0.02 ^a^	5.33 ± 0.07 ^a^
	Amino acid score, AAS (%)	123.23 ± 1.44 ^a^	137.01 ± 2.48	172.45 ± 1.37 ^c^	35.47 ± 5.71	133.22 ± 1.04 ^a^	127.26 ± 0.57 ^a^	106.54 ± 1.31 ^a^
Anterior tendon	Fraction of total amino acid mass (%)	4.86 ± 0.06 ^ab^	9.49 ± 0.12	9.67 ± 0.09 ^ab^	1.51 ± 0.36	7.84 ± 0.09 ^b^	4.99 ± 0.07 ^bcd^	5.15 ± 0.09 ^c^
	Amino acid score, AAS (%)	121.58 ± 1.48 ^ab^	135.54 ± 1.74	175.79 ± 1.63 ^ab^	43.03 ± 10.35	130.70 ± 1.52 ^b^	124.70 ± 1.75 ^bcd^	102.95 ± 1.80 ^c^
Posterior tendon	Fraction of total amino acid mass (%)	4.91 ± 0.06 ^ab^	9.44 ± 0.20	9.67 ± 0.13 ^ab^	1.34 ± 0.45	7.87 ± 0.06 ^ab^	5.02 ± 0.04 ^bc^	5.21 ± 0.06 ^abc^
	Amino acid score, AAS (%)	122.76 ± 1.55 ^ab^	134.92 ± 2.85	175.82 ± 2.35 ^ab^	38.42 ± 12.73	131.17 ± 0.97 ^ab^	125.44 ± 0.95 ^bc^	104.20 ± 1.18 ^abc^

Values represent the mean ± S.D., *n* = 5. Data in the same column are marked with different letters indicating significant differences (*p* < 0.05).

**Table 5:** Fatty acids content of sika deer venison in different muscles.

Category	Fatty Acid	Content (mg/kg)											
		Foreleg	Hind Leg	Outer Tenderloin	Rump	Neck	Chest	Flank	Abdominal Rib	High Rib	Tenderloin	Anterior Tendon	Posterior Tendon
Saturated fatty acid (SFA)	C4:0	21.11 ± 16.46	11.64 ± 12.46	8.43 ± 7.42	41.18 ± 74.23	47.58 ± 69.70	7.77 ± 3.75	9.10 ± 8.16	8.62 ± 5.86	7.70 ± 4.67	4.80 ± 3.36	15.02 ± 19.26	11.96 ± 15.53
	C6:0	9.47 ± 2.55 ^ab^	5.66 ± 3.27 ^bc^	4.27 ± 2.45 ^c^	6.20 ± 3.57 ^bc^	10.29 ± 4.91 ^a^	4.29 ± 1.11 ^c^	5.68 ± 2.55 ^bc^	4.29 ± 2.49 ^c^	5.89 ± 2.46 ^bc^	4.37 ± 2.07 ^c^	7.48 ± 2.42 ^abc^	6.67 ± 2.84 ^abc^
	C8:0	2.35 ± 0.74 ^bc^	1.86 ± 0.64 ^c^	1.66 ± 0.52 ^c^	1.93 ± 0.92 ^bc^	2.95 ± 1.00 ^abc^	2.13 ± 0.48 ^bc^	4.02 ± 2.14 ^a^	3.57 ± 2.42 ^ab^	3.14 ± 1.42 ^abc^	1.81 ± 0.26 ^c^	1.75 ± 0.55 ^c^	1.51 ± 0.24 ^c^
	C10:0	5.28 ± 2.00 ^b^	4.94 ± 2.36 ^b^	5.15 ± 1.99 ^b^	4.99 ± 2.72 ^b^	9.09 ± 3.54 ^b^	8.18 ± 1.96 ^b^	23.02 ± 19.82 ^a^	24.22 ± 16.88 ^a^	11.46 ± 5.12 ^b^	4.58 ± 0.84 ^b^	3.09 ± 1.35 ^b^	3.11 ± 1.51 ^b^
	C12:0	34.67 ± 14.76 ^b^	34.65 ± 18.14 ^b^	35.44 ± 14.87 ^b^	33.53 ± 18.54 ^b^	60.93 ± 27.14 ^b^	56.03 ± 14.30 ^b^	158.64 ± 138.25 ^a^	165.71 ± 120.07 ^a^	75.72 ± 32.33 ^b^	29.68 ± 6.12 ^b^	20.13 ± 10.52 ^b^	21.93 ± 12.35 ^b^
	C13:0	8.86 ± 2.97 ^b^	7.38 ± 3.74 ^b^	6.34 ± 2.61 ^b^	6.70 ± 3.37 ^b^	13.06 ± 6.42 ^b^	10.09 ± 2.48 ^b^	38.57 ± 38.43 ^a^	39.49 ± 27.92 ^a^	16.96 ± 6.49 ^b^	7.58 ± 1.15 ^b^	5.48 ± 2.14 ^b^	6.49 ± 4.11 ^b^
	C14:0	845.94 ± 282.32 ^b^	920.65 ± 484.67 ^b^	914.62 ± 346.05 ^b^	846.92 ± 457.60 ^b^	1497.55 ± 661.44 ^b^	1562.33 ± 520.70 ^b^	3988.40 ± 2810.44 ^a^	4235.60 ± 3197.01 ^a^	2026.27 ± 968.94 ^b^	696.12 ± 115.08 ^b^	437.23 ± 238.02 ^b^	489.06 ± 274.57 ^b^
	C15:0	230.08 ± 56.97 ^b^	189.47 ± 73.53 ^b^	135.33 ± 45.06 ^b^	151.75 ± 61.93 ^b^	270.95 ± 112.01 ^b^	236.25 ± 73.25 ^b^	705.27 ± 553.30 ^a^	752.40 ± 554.55 ^a^	376.50 ± 168.20 ^b^	158.79 ± 18.17 ^b^	125.35 ± 43.52 ^b^	137.90 ± 65.21 ^b^
	C16:0	9651.23 ± 2614.47 ^bcd^	10,971.95 ± 4892.29 ^bcd^	11,699.94 ± 2942.45 ^bc^	7677.75 ± 3993.06 ^bcd^	11,242.19 ± 4508.53 ^bcd^	12,567.07 ± 4326.32 ^b^	22,304.02 ± 7900.55 ^a^	24,116.83 ± 14,693.99 ^a^	12,091.80 ± 3770.68 ^bc^	6275.19 ± 2068.40 ^bcd^	3854.09 ± 1544.93 ^cd^	3156.87 ± 1289.66 ^d^
	C17:0	253.80 ± 64.78 ^c^	188.59 ± 48.54 ^c^	115.97 ± 32.41 ^c^	127.64 ± 37.93 ^c^	245.05 ± 93.98 ^c^	215.52 ± 93.66 ^c^	738.39 ± 632.57 ^ab^	900.79 ± 803.06 ^a^	386.74 ± 213.41 ^bc^	167.98 ± 26.06 ^c^	125.63 ± 40.66 ^c^	129.41 ± 49.46 ^c^
	C18:0	6278.70 ± 1302.94 ^c^	4831.66 ± 855.76 ^c^	20.80 ± 4.81 ^c^	27.86 ± 7.59 ^c^	20.97 ± 3.54 ^c^	6008.86 ± 2528.09 ^c^	17,326.78 ± 15,122.06 ^ab^	21,189.90 ± 18,195.44 ^a^	9521.77 ± 4500.26 ^bc^	4696.18 ± 819.18 ^c^	4357.38 ± 730.57 ^c^	3930.89 ± 1136.47 ^c^
	C20:0	19.69 ± 5.44 ^bc^	15.78 ± 2.82 ^c^	53.71 ± 31.23 ^bc^	67.75 ± 16.35 ^b^	134.33 ± 80.40 ^a^	21.40 ± 9.90 ^bc^	52.61 ± 47.79 ^bc^	69.77 ± 65.46 ^b^	29.46 ± 16.21 ^bc^	11.80 ± 2.68 ^c^	12.13 ± 2.01 ^c^	11.83 ± 4.80 ^c^
	C21:0	9.48 ± 2.25 ^c^	7.36 ± 1.62 ^c^	34.26 ± 10.80 ^b^	52.08 ± 21.52 ^a^	27.59 ± 7.28 ^b^	1.69 ± 0.71 ^c^	3.58 ± 3.80 ^c^	3.96 ± 3.47 ^c^	1.89 ± 0.60 ^c^	0.87 ± 0.26 ^c^	1.06 ± 0.33 ^c^	1.05 ± 0.47 ^c^
	C22:0	23.90 ± 3.87 ^bc^	21.14 ± 2.91 ^bcd^	16.91 ± 3.48 ^bcd^	26.62 ± 9.21 ^b^	41.76 ± 7.44 ^a^	17.34 ± 6.68 ^bcd^	19.93 ± 15.72 ^bcd^	21.57 ± 12.35 ^bcd^	21.21 ± 2.73 ^bcd^	11.86 ± 3.54 ^d^	18.85 ± 4.21 ^bcd^	14.58 ± 2.59 ^cd^
	C23:0	26.91 ± 4.87 ^bc^	26.01 ± 8.55 ^bc^	21.28 ± 2.49 ^bc^	31.15 ± 10.42 ^b^	43.36 ± 6.30 ^a^	21.08 ± 7.74 ^bc^	26.49 ± 19.28 ^bc^	26.50 ± 14.75 ^bc^	31.60 ± 3.85 ^b^	16.51 ± 4.10 ^c^	25.38 ± 4.09 ^bc^	17.87 ± 2.65 ^c^
	C24:0	46.97 ± 5.40 ^ab^	41.87 ± 3.42 ^abc^	31.08 ± 6.06 ^bcde^	50.84 ± 23.29 ^a^	37.07 ± 13.36 ^abcd^	25.36 ± 9.67 ^cde^	28.85 ± 20.63 ^cde^	31.24 ± 17.09 ^bcde^	32.15 ± 5.60 ^bcde^	19.17 ± 6.18 ^e^	28.69 ± 4.83 ^cde^	21.68 ± 3.71 ^de^
	Total	17,458.33 ± 3583.76 ^b^	17,280.61 ± 6216.30 ^b^	13,105.18 ± 3336.00 ^b^	9154.90 ± 4351.50 ^b^	13,704.71 ± 5387.21 ^b^	20,765.38 ± 6593.54 ^b^	45,433.36 ± 25,742.66 ^a^	51,594.45 ± ± 36,214.34 ^a^	24,640.26 ± 8367.23 ^b^	12,107.30 ± 1350.28 ^b^	9038.75 ± 2378.78 ^b^	7962.80 ± 2576.07 ^b^
Monounsaturated fatty acid (MUFA)	C14:1, cis-9	352.04 ± 141.29 ^c^	439.14 ± 213.51 ^bc^	315.16 ± 120.92 ^c^	461.70 ± 287.64 ^bc^	618.70 ± 269.42 ^abc^	1018.88 ± 370.71 ^abc^	1227.14 ± 692.63 ^ab^	1335.74 ± 1821.87 ^a^	759.46 ± 339.52 ^abc^	205.69 ± 38.89 ^c^	242.32 ± 163.59 ^c^	251.98 ± 89.52 ^c^
	C15:1, cis-10	0.00 ± 0.00 ^d^	0.00 ± 0.00 ^d^	5.04 ± 2.29 ^c^	9.12 ± 5.90 ^b^	12.42 ± 5.68 ^a^	0.00 ± 0.00 ^d^	0.00 ± 0.00 ^d^	0.00 ± 0.00 ^d^	0.00 ± 0.00 ^d^	0.00 ± 0.00 ^d^	0.00 ± 0.00 ^d^	0.00 ± 0.00 ^d^
	C16:1, cis-9	4091.99 ± 1640.50 ^bc^	5254.87 ± 2596.23 ^bc^	4005.69 ± 986.63 ^bc^	3929.23 ± 3076.57 ^bc^	4614.36 ± 1938.37 ^bc^	13,928.18 ± 7565.44 ^ab^	16,764.52 ± 9336.03 ^a^	16,875.33 ± 20,405.46 ^a^	9945.67 ± 5771.93 ^abc^	2542.52 ± 449.23 ^c^	2932.14 ± 1872.01 ^c^	2841.64 ± 824.50 ^c^
	C17:1, cis-10	0.00 ± 0.00 ^d^	0.00 ± 0.00 ^d^	53.33 ± 31.98 ^cd^	84.95 ± 35.46 ^cd^	146.03 ± 53.18 ^cd^	174.39 ± 79.04 ^bc^	297.39 ± 153.14 ^ab^	346.11 ± 290.72 ^a^	199.60 ± 115.98 ^bc^	64.48 ± 12.17 ^cd^	69.21 ± 33.05 ^cd^	63.54 ± 13.26 ^cd^
	C18:1, cis-9	6671.29 ± 1935.77 ^c^	5526.38 ± 1627.34 ^c^	4867.17 ± 1236.53 ^c^	4109.46 ± 1918.92 ^c^	6303.73 ± 2386.82 ^c^	14,380.67 ± 9127.01 ^bc^	25,942.02 ± 13,495.62 ^ab^	31,772.73 ± 27,495.22 ^a^	8657.01 ± 5127.39 ^c^	4857.99 ± 1200.72 ^c^	4873.26 ± 2455.81 ^c^	4536.96 ± 713.46 ^c^
	C20:1, cis-11	97.31 ± 81.88 ^bc^	68.39 ± 16.30 ^bc^	35.10 ± 7.60 ^c^	48.73 ± 13.38 ^bc^	70.97 ± 7.91 ^bc^	214.28 ± 201.13 ^abc^	268.95 ± 112.80 ^ab^	377.18 ± 416.20 ^a^	199.97 ± 218.46 ^abc^	41.20 ± 21.72 ^bc^	57.65 ± 48.51 ^bc^	50.75 ± 30.12 ^bc^
	C22:1, cis-13	0.00 ± 0.00 ^c^	0.00 ± 0.00 ^c^	0.00 ± 0.00 ^c^	0.00 ± 0.00 ^c^	0.00 ± 0.00 ^c^	8.79 ± 6.98 ^abc^	12.63 ± 10.32 ^ab^	17.13 ± 15.65 ^a^	17.33 ± 17.55 ^a^	3.79 ± 0.70 ^bc^	5.14 ± 2.11 ^bc^	3.77 ± 1.36 ^bc^
	C24:1	0.00 ± 0.00 ^c^	0.00 ± 0.00 ^c^	0.00 ± 0.00 ^c^	0.00 ± 0.00 ^c^	0.00 ± 0.00 ^c^	0.00 ± 0.00 ^c^	0.00 ± 0.00 ^c^	0.00 ± 0.00 ^c^	0.00 ± 0.00 ^c^	0.00 ± 0.00 ^c^	7.47 ± 2.28 ^a^	4.82 ± 0.86 ^b^
	Total	11,212.63 ± 3511.50 ^c^	11,288.77 ± 4266.01 ^c^	9281.49 ± 2229.36 ^c^	8643.18 ± 4990.82 ^c^	11,766.21 ± 4590.53 ^c^	29,725.18 ± 17,017.45 ^bc^	44,512.66 ± 20,492.99 ^ab^	50,724.21 ± 45,542.10 ^a^	19,779.04 ± 3353.53 ^c^	7715.67 ± 1513.31 ^c^	8187.18 ± 4493.81 ^c^	7753.47 ± 1615.34 ^c^
Polyunsaturated fatty acid (PUFA)	Linoleic acid, C18:2, cis-9, 12 *ω-6*	8212.50 ± 491.66 ^abcd^	6631.45 ± 874.65 ^abcd^	4258.24 ± 514.77 ^cd^	3829.20 ± 1014.51 ^cd^	3572.59 ± 850.81 ^d^	6514.38 ± 1506.26 ^abcd^	9831.04 ± 9666.60 ^ab^	11,028.78 ± 6487.24 ^a^	8883.91 ± 749.77 ^abc^	6908.19 ± 1634.94 ^abcd^	6687.91 ± 1301.81 ^abcd^	5426.19 ± 1123.06 ^bcd^
	*γ*-linolenic acid, C18:3, cis-6, 9, 12 *ω-6*	42.84 ± 6.46 ^bc^	43.75 ± 5.41 ^b^	56.55 ± 12.18 ^b^	78.95 ± 19.79 ^a^	89.60 ± 23.84 ^a^	22.16 ± 4.65 ^d^	23.71 ± 16.64 ^d^	26.17 ± 15.16 ^d^	27.22 ± 5.00 ^cd^	24.69 ± 4.06 ^d^	23.15 ± 7.13 ^d^	20.42 ± 4.27 ^d^
	*α*-linolenic acid, C18:3, cis-9, 12, 15 *ω-3*	143.68 ± 50.89 ^abc^	110.57 ± 15.61 ^abcd^	10.34 ± 2.68 ^d^	12.54 ± 4.13 ^d^	22.36 ± 5.99 ^d^	82.79 ± 20.82 ^bcd^	167.57 ± 174.38 ^ab^	196.58 ± 142.75 ^a^	109.14 ± 28.09 ^abcd^	75.12 ± 14.66 ^bcd^	69.29 ± 16.29 ^bcd^	57.43 ± 9.53 ^cd^
	C20:2, cis-11, 14	86.86 ± 36.57 ^cde^	60.06 ± 5.55 ^de^	118.19 ± 23.56 ^bcd^	171.03 ± 50.33 ^ab^	178.62 ± 24.52 ^a^	64.75 ± 24.12 ^de^	91.86 ± 68.60 ^cde^	124.11 ± 92.13 ^abc^	86.19 ± 32.95 ^cde^	49.54 ± 14.43 ^e^	54.96 ± 11.55 ^e^	40.70 ± 6.87 ^e^
	C20:3, cis-8, 11, 14	230.82 ± 49.94 ^a^	211.42 ± 35.58 ^ab^	9.78 ± 2.02 ^c^	13.09 ± 4.51 ^c^	13.16 ± 2.03 ^c^	178.35 ± 63.40 ^ab^	187.55 ± 142.93 ^ab^	214.13 ± 131.41 ^ab^	208.14 ± 38.60 ^ab^	160.81 ± 57.61 ^ab^	176.04 ± 20.51 ^ab^	131.76 ± 30.28 ^b^
	C20:3, cis-11, 14, 17	18.19 ± 2.67 ^bc^	19.04 ± 4.16 ^abc^	13.86 ± 2.71 ^bc^	20.89 ± 7.27 ^ab^	28.52 ± 4.53 ^a^	12.06 ± 3.06 ^bc^	16.84 ± 17.47 ^bc^	20.67 ± 13.89 ^ab^	14.89 ± 3.26 ^bc^	9.84 ± 2.73 ^c^	9.81 ± 2.31 ^c^	8.91 ± 1.60 ^c^
	C20:4, cis-5, 8, 11, 14 *ω-6*	2867.20 ± 386.03 ^a^	2981.98 ± 436.29 ^a^	1949.03 ± 175.52 ^ab^	1781.24 ± 396.24 ^ab^	1369.43 ± 308.79 ^b^	2882.34 ± 917.20 ^a^	2577.10 ± 2354.80 ^ab^	2953.16 ± 1734.88 ^a^	3177.09 ± 617.15 ^a^	2690.32 ± 908.32 ^ab^	2946.42 ± 368.45 ^a^	2420.90 ± 645.27 ^ab^
	C20:5, cis-5, 8, 11, 14, 17 *ω-3*	45.30 ± 3.52 ^b^	60.92 ± 17.48 ^a^	5.38 ± 3.11 ^e^	5.61 ± 3.00 ^e^	11.17 ± 4.79 ^de^	36.79 ± 13.32 ^bc^	25.57 ± 22.89 ^cd^	26.32 ± 17.00 ^cd^	32.18 ± 10.23 ^bc^	35.06 ± 13.08 ^bc^	29.02 ± 7.75 ^bc^	28.91 ± 10.01 ^bc^
	C22:6, cis-4, 7, 10, 13, 16, 19 *ω-3*	59.78 ± 17.52 ^ab^	65.23 ± 22.05 ^a^	3.61 ± 0.43 ^d^	5.85 ± 3.07 ^d^	9.76 ± 1.80 ^cd^	37.10 ± 20.93 ^b^	36.28 ± 31.86 ^b^	41.30 ± 27.26 ^ab^	42.47 ± 20.49 ^ab^	33.14 ± 18.81 ^bc^	38.83 ± 8.48 ^b^	34.20 ± 13.51 ^bc^
	Total	11,707.18 ± 354.78 ^abcd^	10,184.42 ± 1080.65 ^abcd^	6424.99 ± 686.82 ^bcd^	5918.40 ± 1362.56 ^cd^	5295.21 ± 1113.21 ^d^	9830.72 ± 2474.41 ^abcd^	12,957.53 ± 12,429.14 ^ab^	14,631.23 ± 8418.25 ^a^	12,581.24 ± 1267.22 ^abc^	9986.72 ± 2598.98 ^abcd^	10,035.44 ± 1629.26 ^abcd^	8169.44 ± 1806.23 ^abcd^
	Total fatty acids (TFA)	40,388.26 ± 7141.13 ^b^	38,753.80 ± 10,326.94 ^b^	28,811.66 ± 5525.24 ^b^	23,716.47 ± 10,292.26 ^b^	30,766.14 ± 10,036.79 ^b^	60,321.28 ± 23,566.19 ^b^	102,903.55 ± 55,763.87 ^a^	116,949.90 ± 84,667.09 ^a^	57,000.54 ± 10,390.53 ^b^	29,809.69 ± 2660.97 ^b^	27,261.38 ± 7735.46 ^b^	23,885.71 ± 3821.79 ^b^

Values represent the mean ± S.D., *n* = 5. Data in the same column are marked with different letters indicating significant differences (*p* < 0.05).

**Table 6:** Essential fatty acid index of sika deer venison in different muscles.

Index	Content (mg/kg)											
	Foreleg	Hind Leg	Outer Tenderloin	Rump	Neck	Chest	Flank	Abdominal Rib	High Rib	Tenderloin	Anterior Tendon	Posterior Tendon
EFA (Linoleic acid + *α*-linolenic acid)	8356.18 ± 492.86 ^abcd^	6742.02 ± 886.38 ^abcd^	4268.59 ± 516.17 ^cd^	3841.74 ± 1013.95 ^cd^	3594.95 ± 855.07 ^d^	6597.17 ± 1523.03 ^abcd^	9998.61 ± 9840.28 ^ab^	11,225.37 ± 6618.14 ^a^	8993.05 ± 768.03 ^abc^	6983.31 ± 1647.41 ^abcd^	6757.20 ± 1316.40 ^abcd^	5483.62 ± 1132.08 ^bcd^
∑*ω*-3	248.76 ± 52.54 ^ab^	236.71 ± 26.05 ^abc^	19.33 ± 5.67 ^f^	24.00 ± 9.17 ^ef^	43.29 ± 11.27 ^def^	156.67 ± 45.78 ^abcd^	229.42 ± 216.11 ^abc^	264.21 ± 163.58 ^a^	183.80 ± 32.48 ^abc^	143.32 ± 42.01 ^abcd^	137.14 ± 20.11 ^bcde^	120.54 ± 30.16 ^cdef^
∑*ω*-6	11,122.54 ± 474.40 ^abcd^	9657.19 ± 1045.02 ^abcd^	6263.82 ± 666.32 ^bcd^	5689.39 ± 1404.78 ^cd^	5031.62 ± 1097.16 ^d^	9418.88 ± 2347.39 ^abcd^	12,431.85 ± 11,989.23 ^ab^	14,008.12 ± 8035.46 ^a^	12,088.22 ± 1188.38 ^abc^	9623.21 ± 2495.96 ^abcd^	9657.48 ± 1609.20 ^abcd^	7867.51 ± 1747.81 ^abcd^
*ω*-6/*ω*-3	46.27 ± 9.42 ^b^	40.92 ± 3.59 ^b^	346.11 ± 104.79 ^a^	278.51 ± 161.65 ^a^	120.79 ± 32.74 ^b^	60.96 ± 5.10 ^b^	54.74 ± 8.15 ^b^	55.65 ± 8.18 ^b^	66.56 ± 5.93 ^b^	67.76 ± 5.83 ^b^	70.28 ± 4.27 ^b^	65.91 ± 4.84 ^b^
PUFA/SFA	0.69 ± 0.14 ^cd^	0.66 ± 0.28 ^cd^	0.53 ± 0.19 ^cde^	0.73 ± 0.26 ^cd^	0.45 ± 0.22 ^de^	0.50 ± 0.16 ^de^	0.26 ± 0.10 ^e^	0.32 ± 0.11 ^e^	0.56 ± 0.20 ^cde^	0.84 ± 0.26 ^bc^	1.16 ± 0.25 ^a^	1.10 ± 0.38 ^ab^
EFA/TFA/%	21.10 ± 3.02 ^abc^	18.28 ± 4.53 ^bcd^	15.19 ± 2.92 ^def^	17.38 ± 4.17 ^cde^	12.65 ± 4.42 ^efg^	11.88 ± 3.93 ^fg^	8.72 ± 3.87 ^g^	11.09 ± 3.84 ^fg^	16.08 ± 2.50 ^cdef^	23.27 ± 4.13 ^ab^	25.52 ± 3.74 ^a^	23.01 ± 3.50 ^ab^

Values represent the mean ± S.D., *n* = 5. Data in the same column are marked with different letters indicating significant differences (*p* < 0.05).

**Table 7:** Squalene content of sika deer venison in different muscles.

Content	Content (mg/kg)									
	Outer Tenderloin	Rump	Neck	Chest	Flank	Abdominal rib	High Rib	Tenderloin	Anterior Tendon	Posterior Tendon
Squalene	34.63 ± 4.47 ^d^	40.19 ± 12.61 ^cd^	30.13 ± 8.07 ^d^	74.14 ± 13.09 ^abc^	93.25 ± 64.83 ^ab^	100.85 ± 51.77 ^a^	58.67 ± 11.53 ^bcd^	66.56 ± 14.95 ^abcd^	51.09 ± 13.02 ^cd^	57.03 ± 8.22 ^bcd^

Values represent the mean ± S.D., *n* = 5. Data in the same column are marked with different letters indicating significant differences (*p* < 0.05).

## Data Availability

The data used to support the findings of this study can be made available by the corresponding author upon request.
